# Human magnetic sense is mediated by a light and magnetic field resonance-dependent mechanism

**DOI:** 10.1038/s41598-022-12460-6

**Published:** 2022-05-30

**Authors:** Kwon-Seok Chae, Soo-Chan Kim, Hye-Jin Kwon, Yongkuk Kim

**Affiliations:** 1 Department of Biology Education, Kyungpook National University, Daegu, 41566 Republic of Korea; 2 Department of Nanoscience and Nanotechnology, Kyungpook National University, Daegu, 41566 Republic of Korea; 3 Brain Science and Engineering Institute, Kyungpook National University, Daegu, 41566 Republic of Korea; 4 Department of Electrical and Electronic Engineering, Research Center for Applied Human Sciences, Hankyong National University, Anseong, 17579 Republic of Korea; 5 Department of Mathematics, Kyungpook National University, Daegu, 41566 Republic of Korea

**Keywords:** Ecology, Neuroscience, Physiology, Zoology

## Abstract

Numerous organisms use the Earth’s magnetic field as a sensory cue for migration, body alignment, or food search. Despite some contradictory reports, yet it is generally accepted that humans do not sense the geomagnetic field. Here, we demonstrate that a magnetic field resonance mechanism mediates light-dependent magnetic orientation in men, using a rotary chair experiment combined with a two-alternative forced choice paradigm. Two groups of subjects were classified with different magnetic orientation tendencies depending on the food context. Magnetic orientation of the subjects was sensitive to the wavelength of incident light and was critically dependent on blue light reaching the eyes. Importantly, it appears that a magnetic field resonance-dependent mechanism mediates these responses, as evidenced by disruption or augmentation of the ability to orient by radiofrequency magnetic fields at the Larmor frequency and the dependence of these effects on the angle between the radiofrequency and geomagnetic fields. Furthermore, inversion of the vertical component of the geomagnetic field revealed a non-canonical inclination compass effect on the magnetic orientation. These results establish the existence of a human magnetic sense and suggest an underlying quantum mechanical magnetoreception mechanism.

## Introduction

Numerous organisms, across a wide range of taxa, including birds, sea turtles, reptiles, insects, magnetotactic bacteria, and plants, use the geomagnetic field (GMF) as a sensory cue for migration^1–4^, short-distance movement^5–7^, body alignment^8–10^, food search^6,7^, or growth (plants)^11^, depending on the species and biological context. Both magnetic compass^2,12^ and magnetic map^3,13^ information can be derived from the GMF; the former being essential for a variety of magnetically sensitive behaviors^5–10,12,14,15^. An inclination^12,16^ or polarity compass^17,18^ may provide animals with directional information by contrasting mechanisms: light-dependent radical pairs^19–21^ in cryptochrome flavoproteins in the eyes of birds^20,21^ and light-independent iron-containing biogenic magnetite in bacteria or the ethmoid bone of salmon^14,22^. It is thought that light-induced radical pairs, comprised of a flavin adenine dinucleotide (FAD) radical and a tryptophan radical, in cryptochromes act as the magnetic compass sensor in migratory birds through a quantum mechanical mechanism^20,21,23^. In these species, the inclination compass is activated by blue or green light^24^ and disrupted by red light^25^, indicating that different wavelengths play different roles in radical pair-mediated behaviors^21^.

Research on magnetoreception in humans is very limited. It is widely accepted that the Earth’s static magnetic field is not sensed by humans, while alternating magnetic fields, such as power frequency fields^26^ and pulsed fields^27^, can have adverse health effects and therapeutic applications, respectively. Following previous controversial reports^28–30^, two recent studies, using different experimental approaches, support the existence of GMF responses in humans^31,32^ with a sharp contrast. In a rotary chair experiment, starved men but not women were able to orient in a blue-light-dependent manner towards a particular magnetic direction that had previously been associated with food in the ambient GMF^31^. This study suggests that the magnetoreceptors are in the eyes but does not demonstrate the underlying sensory mechanism. In contrast, electroencephalography showed that a decrease in alpha brain wave activity occurred in some human subjects under conditions of darkness^32^. The observed sensitivity to the polarity of the applied magnetic fields implied a magnetite-based mechanism. However, so far, the existence of a human magnetic sense itself and any underlying mechanism are far from clear.

To establish the existence and underlying mechanism of human magnetic sensing, we have studied magnetic orientation in men, which showed remarkable magnetic sensitivity, by combining the rotary chair method with a two-alternative forced choice (2-AFC) paradigm^33,34^, applying oscillating magnetic fields as a diagnostic tool for a magnetic field resonance mechanism, such as the radical pair mechanism^35^.

## Results

### Differential sensitivity in ambient GMF-responsive orientation

**Figure 1:**
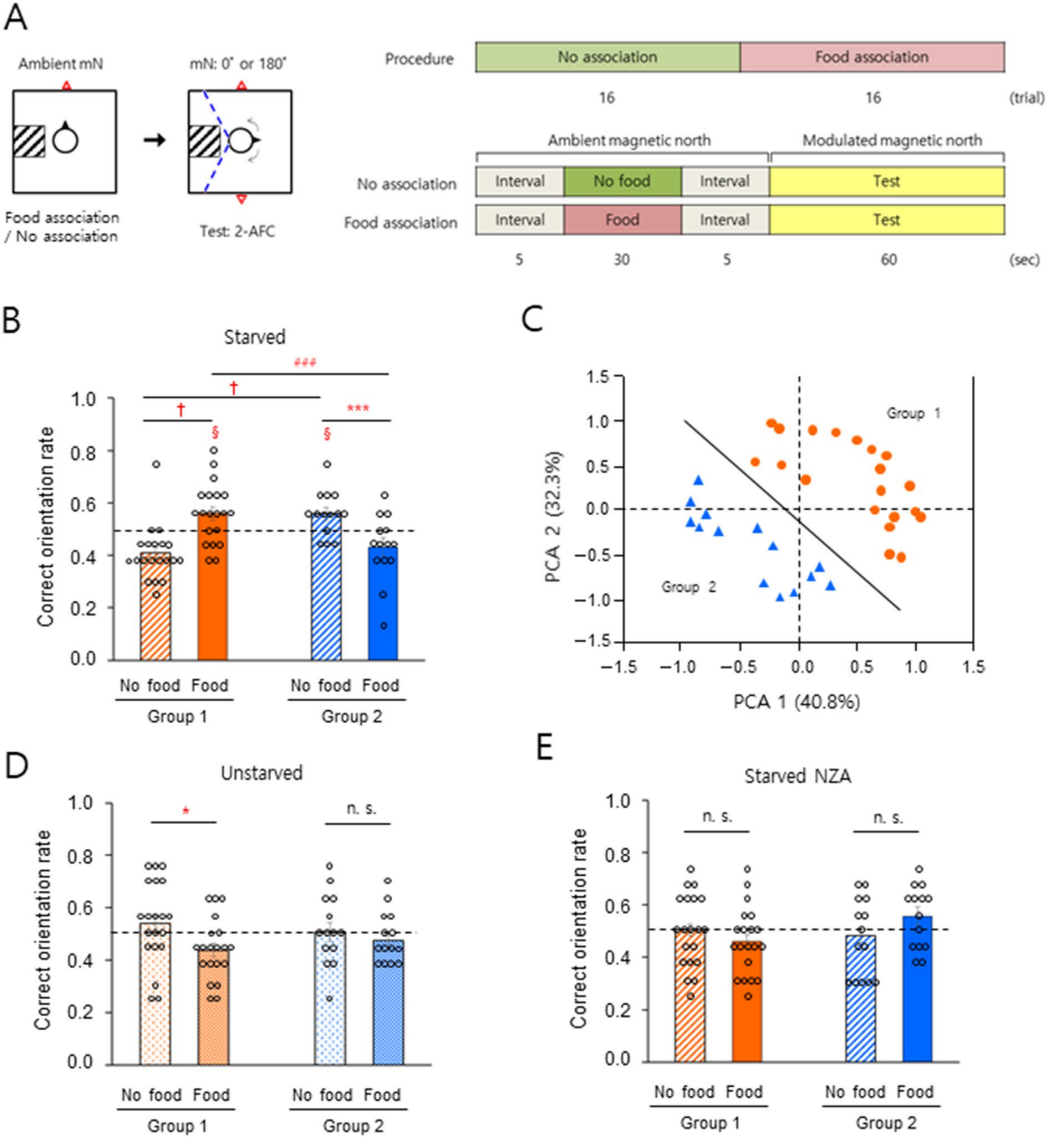
Different sensitivity of geomagnetic orientation in men. (**A**) Schematic drawing of a 2-AFC behavioral paradigm to test geomagnetic orientation in men. *Left*: Top view of the subject facing toward the ambient geomagnetic north during the no-association/food-association phase, and rotating to search for one of the two modulated magnetic norths randomly provided during the test phase. Subjects were allowed to rotate clockwise or counterclockwise during the test as shown by arrows within the range indicated by the dashed blue lines (see “Methods” section). mN, magnetic north; square, the vertical Helmholtz coils; square with hatched lines, stool to limit the subject’s rotation; circle, the subject; black closed triangle, the facing direction of the subject; red open triangle(s), direction of the ambient magnetic north (association phase) or modulated magnetic norths (test phase). *Right*: The procedure and timeline for the 2-AFC experiment. Food was provided for ‘food-association’ trials only. (**B**) A significant increase (Group 1) and decrease (Group 2) in correct orientation rate between the different associations under starved conditions. (**C**) Two subject groups were classified by principal component analysis. Analyzed parameters; correct orientation rate with the food/no-food association under light wavelength of 350–800 nm or 400–800 nm (data, *n* = 136; 34 subjects). Group 1 (orange filled circle), *n* = 20; Group 2 (blue filled triangle), *n* = 14. Solid line, the classifying boundary (PCA 2 =  − 1.1515 PCA 1 − 0.0897) (**D**) A significant decrease (Group 1) in correct orientation rate by the change of association under the unstarved condition. (**E**) No notable change of correct orientation rate with near-zero GMF during the association phase. NZA, near-zero GMF association; §, *P* < 0.05 by one-sample *t-*test; *, *P* < 0.05; ***, *P* < 0.005 and n.s., not significant by paired sample *t-*test; ^###^, *P* < 0.005 by two sample *t-*test; †, *P*-value, 0.9999 > 0.9750 (confidence interval, − 0.2170, − 0.0820) for the data of no-association and food-association in Group 1, and 1.0000 > 0.9750 (confidence interval, − 0.0801, 0.0786) for the data of no-association in Group 1 and Group 2 by a percentile bootstrap analysis (see Fig. S2); horizontal dashed lines, 0.5 for correct orientation rate; error bars, standard error of the mean (SEM).

**Figure 2:**
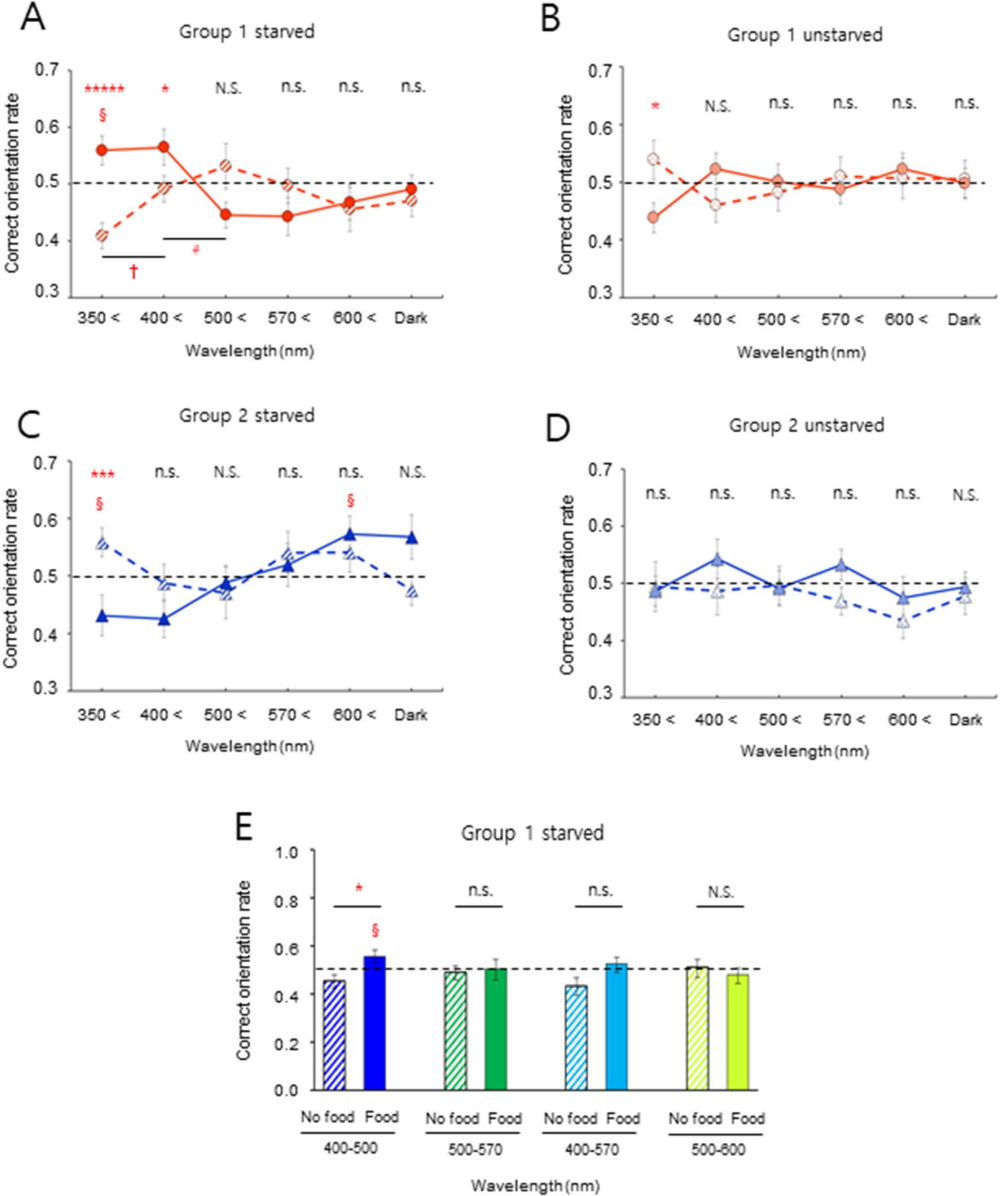
Blue light plays a pivotal role in wavelength-dependent magnetic orientation. (**A**–**D**) Correct magnetic orientation rate in Group 1 (**A**, **B**) or Group 2 (**C**, **D**) subjects under starved and unstarved conditions. The data for > 350 nm light were the same as those in Fig. 1, B or D. Data points connected by straight lines and dashed lines were obtained from food-association and no-association, respectively. §, *P* < 0.05 by one-sample *t-*test; *, *P* < 0.05; ***, *P* < 0.005; *****, *P* < 0.0005; and n.s., not significant by paired sample *t-*test between the corresponding data from food- and no-association; #, *P* < 0.01 by paired sample *t-*test between the data from > 400 nm and > 500 nm in food-association. (**E**) Correct magnetic orientation rate with the different light conditions in Group 1 starved. §, *P* < 0.05 by one-sample *t-*test; *, *P* < 0.05; and n.s., not significant by paired sample *t-*test; †, significant between the data from > 350 nm and > 400 nm in no-association; N. S., not significant by a percentile bootstrap analysis (see Appendixes S1 and S2); horizontal dashed lines, 0.5 for correct orientation rate; error bars, standard error of the mean (SEM). Group 1, *n* = 20 and Group 2, *n* = 14.

Male subjects were starved or fed normally, then tested using the rotary chair method combined with a 2-AFC paradigm that required the subjects to choose one of the two directions on the magnetic north–south axis (Fig. 1A, see “Methods” section). During the association phase of the experiment, subjects with eyes closed and wearing earmuffs were aligned by experimenters so as to face ambient magnetic north while sitting on a rotatable chair and were either conditioned to associate this direction with food or were not conditioned, depending on the session. During the test phase, in which the ‘modulated’ magnetic north was randomly set to true magnetic north or true magnetic south, subjects were asked to indicate the modulated magnetic north direction, without reference to other information including visual and auditory cues. The test was initially performed under full-wavelength visible light (350–800 nm) (Table S1, #1 and Fig. S1A). Subjects that had been starved to produce a significant reduction in blood glucose levels (Table S2) were divided into two groups. Group 1 (Fig. 1B, *left*, *n* = 20, ca. 60%) showed a significant increase in the ability to orient correctly with food-association compared to subjects who had not been conditioned while Group 2 (Fig. 1B, *right*, *n* = 14, ca. 40%) showed a significantly lower rate of food-association (see Figs. S2 and 1C for clustering of the subjects). In our previous study^31^, under full wavelength and > 400 nm but not > 500 nm light conditions, a majority of subjects showed significantly correct magnetic orientations with food-association, whereas the remaining did not. In an attempt to objectively cluster the subjects in the present study, principal component analysis^36,37^ of data recorded for starved subjects with and without food-association under the full wavelength and > 400 nm light conditions as shown in Fig. 2A,C, produced two subject groups (Fig. 1C). The results shown in Fig. 1B are consistent with a reanalysis of relevant data from our previous study^31^ (Fig. S3) and indicate that starved men with food-association were clustered into two groups; one group that showed significant GMF-orientation toward modulated magnetic north on the magnetic north–south axis, and one that did not. By contrast, when the subjects were normally fed (Table S2), Group 1 but not Group 2 showed a significant decrease in correct orientation rate with food-association (Fig. 1D). Subjects were then tested to identify modulated magnetic north randomly set on the true magnetic east–west axis. Similar to the results in Fig. 1B, the orientation rate with food-association increased in Group 1 and decreased in Group 2, compared to no-association with starvation (Fig. S4, Table S2). In our previous study, the ambient GMF was thought to be an unconditioned stimulus during the association phase^31^. Therefore, we examined whether sensing the ambient GMF during association was necessary for correct magnetic orientation on the magnetic north–south axis, as shown in Fig. 1B. Under a near-zero GMF condition during the association phase, neither starved group demonstrated a significant difference in correct orientation rate with and without food association (Fig. 1E), indicating that sensing the ambient GMF prior to the test was necessary for correct magnetic orientation in both groups. These results show that there are two groups with different magnetic sensitivity in ambient GMF-orientation, depending on the food context.

### Pivotal role of blue light in wavelength-dependent distinct magnetic orientation

Based on our previous study^31^ which showed that light was necessary for food-associated geomagnetic orientation in starved men, the effect of wavelength on magnetic orientation was investigated in the two groups. Distinct magnetic orientation profiles that were highly dependent on food context and Group were obtained under six light conditions: five different light wavelengths (Table S1, #1–5 and Fig. S1A–E) and ‘dark’ (Fig. 2A–D). The starved subjects in Group 1 showed significant correct orientation rate with food-association compared to no-association under full wavelength and > 400 nm light, while the orientation rates were dramatically reversed under > 500 nm light (Fig. 2A). This result indicates that UV-A–blue (350–500 nm) and green (500–570 nm) light are either necessary for, or inhibit, correct magnetic orientation and that these effects occur in different directions for the two wavelength ranges depending on the association conditions. This finding is reminiscent of the antagonistic effect of < 450 nm and > 500 nm light on magnetic orientation in newts and flies^38^. The unstarved Group 1 subjects showed a low orientation rate under > 400 nm light with no-association, suggesting that UV-A light (350–400 nm) was helpful for correct orientation under the full wavelength whereas blue light (400–500 nm) decreased the orientation rate (Fig. 2B). By contrast, Group 2 subjects demonstrated no significant differences in orientation rate for changes of wavelength and association conditions, with or without starvation (Fig. 2C,D). However, there was a significant change in the rate for > 400 nm light compared to the full wavelength range, wherein the effect of UV-A light was not negligible and there was a notable rate under red light (> 600 nm) with food-association (Fig. 2C). There were no significant differences between orientation rates in the dark condition by association, starvation, or group, except for a slightly higher rate in starved Group 2 with food-association, indicating that light was crucial for correct magnetic orientation. To dissect the interplay among light wavelengths, blue light alone was sufficient to increase the rate in starved Group 1 subjects (Fig. 2E), suggesting that together with the data in Fig. 2A red light disrupted an increase in the rate elicited by green light, but not by blue light, with the food-association. Importantly, this result indicates that blue light plays a pivotal role in magnetic orientation by overcoming the effect of red light. In addition, UV-A light alone produced a distinct magnetic orientation profile, depending on the group and food context (Fig. S5). These results demonstrate that human geomagnetic orientation is highly sensitive to light wavelength, and that blue light plays a critical role in orientation.

### A magnetic field resonance mechanism-mediated human magnetic orientation

**Figure 3:**
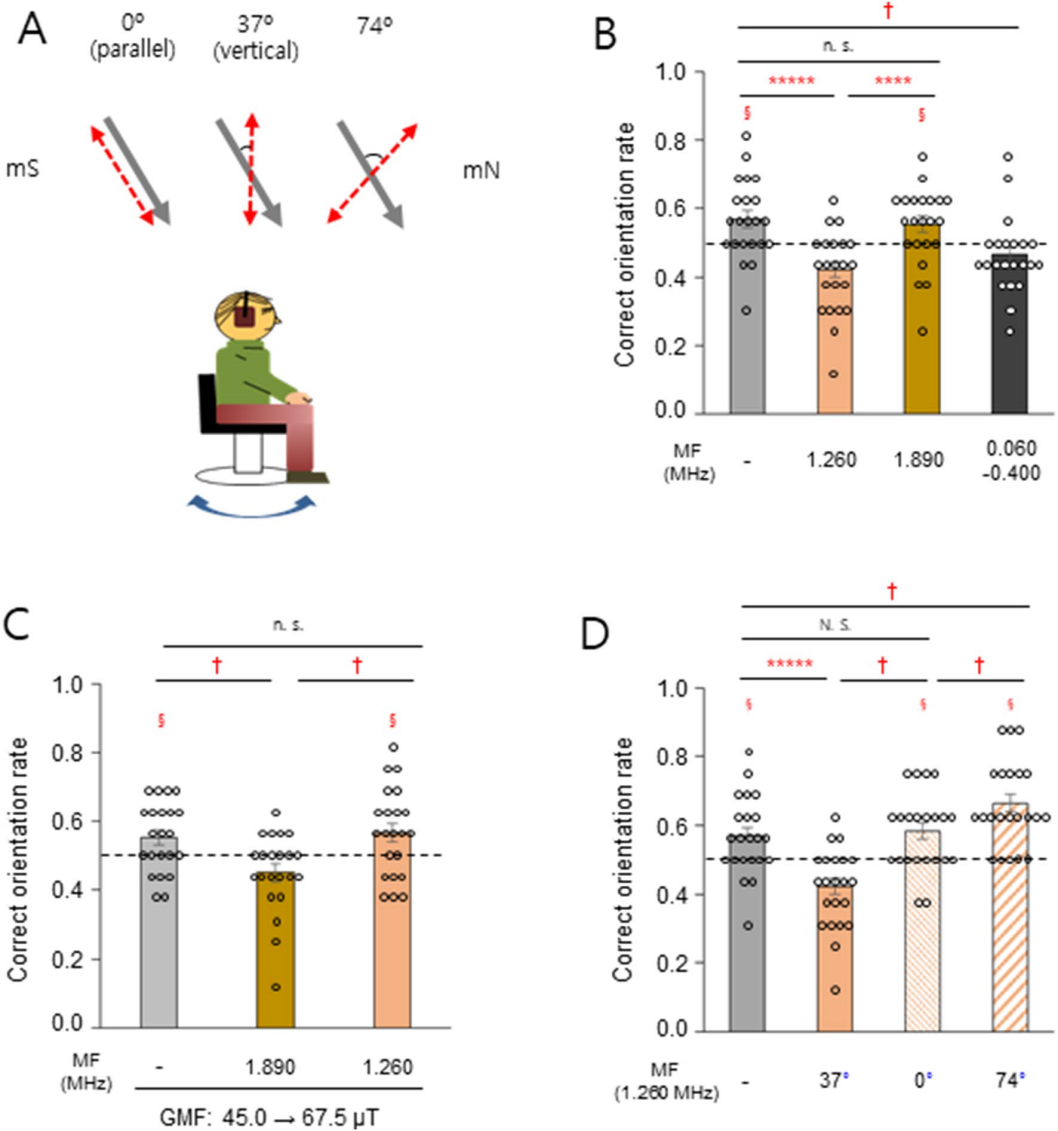
Radical pair mechanism mediates magnetic orientation in humans. (**A**) Experimental set-up to test oscillating magnetic field (red dashed arrows) effect on magnetic orientation of the subjects. The selected starved subjects mentioned in the main text were food/no-food associated and tested under the ambient GMF (45.0 μT, gray arrows) with full wavelength light. (**B**) Correct orientation rate of the subjects exposed to oscillating magnetic fields of different frequency. (**C**) Subjects were tested under an increased intensity of the GMF (67.5 μT), while the ambient GMF was maintained during the association phase. (**D**) Different directions of the 1.260 MHz magnetic field relative to the ambient GMF; 0° (parallel), 37° (vertical), and 74°. Note that in the parallel and 74° configurations, the 1.260 MHz magnetic field had the same angle with respect to the subjects. For 0° and 74° angles, the radiofrequency field was provided only in trials when modulated magnetic north was set to true magnetic north (mN, 0°), but not true magnetic south (mS, 180°), and the correct orientation rate was calculated on these trials alone. §, *P* < 0.05 by one-sample *t-*test; ****, *P* < 0.001; *****, *P* < 0.0005; and n.s., not significant by paired sample *t-*test; †, significant; N. S., not significant by a percentile bootstrap analysis (see Appendixes S1 and S2); horizontal dashed lines, 0.5 for correct orientation rate; error bars, standard error of the mean (SEM). Subjects, *n* = 22.

Blue light is essential for radical pair-mediated magnetoreception in birds^21,39^. Therefore, to understand the magnetosensory mechanism in humans, we tested whether oscillating magnetic fields at particular frequencies^35,40,41^ disrupt magnetic orientation in humans. First, 1.260 MHz, the electron Larmor frequency^21^ in the ambient GMF (45.0 μT), or a flanking frequency (1.890 MHz or 60–400 kHz)^39,40^ was provided vertically to the starved subjects who had shown a correct orientation rate greater than 0.5 in Fig. 1B (Group 1 with food-association and Group 2 with no-association, Fig. 3A, *middle* and Fig. S6). The 1.260 MHz and lower frequency broadband magnetic fields, but not the 1.890 MHz field, significantly disrupted magnetic orientation compared to the dummy load control (Fig. 3B). When the strength of the static magnetic field was increased by 50% to 67.5 μT, such that the Larmor frequency was 1.890 MHz, we found that the 1.890 MHz field, but not the 1.260 MHz field, significantly reduced the correct orientation rate (Fig. 3C). Given the critical property of the proposed radical pair magnetoreceptor as a direction sensor^20,21^, the effect of the angle between the 1.260 MHz field and the ambient GMF was evaluated (Fig. 3A, *left* and *right*). The different orientations of the radiofrequency antenna produced different magnetic field intensities at the eyes of the subjects, caused by the shadowing effects^42^ of their heads. (Table S3). Compared to the dummy load control, the rate was not notably changed in the parallel configuration (0°), whereas it was significantly decreased in the vertical arrangement (37°) and increased for the 74° angle, indicating a differential effect on an anisotropic receptor (Fig. 3D). These results support the notion that radical pairs probably mediate the magnetic orientation observed in humans.

### A non-canonical magnetic inclination compass in humans

**Figure 4:**
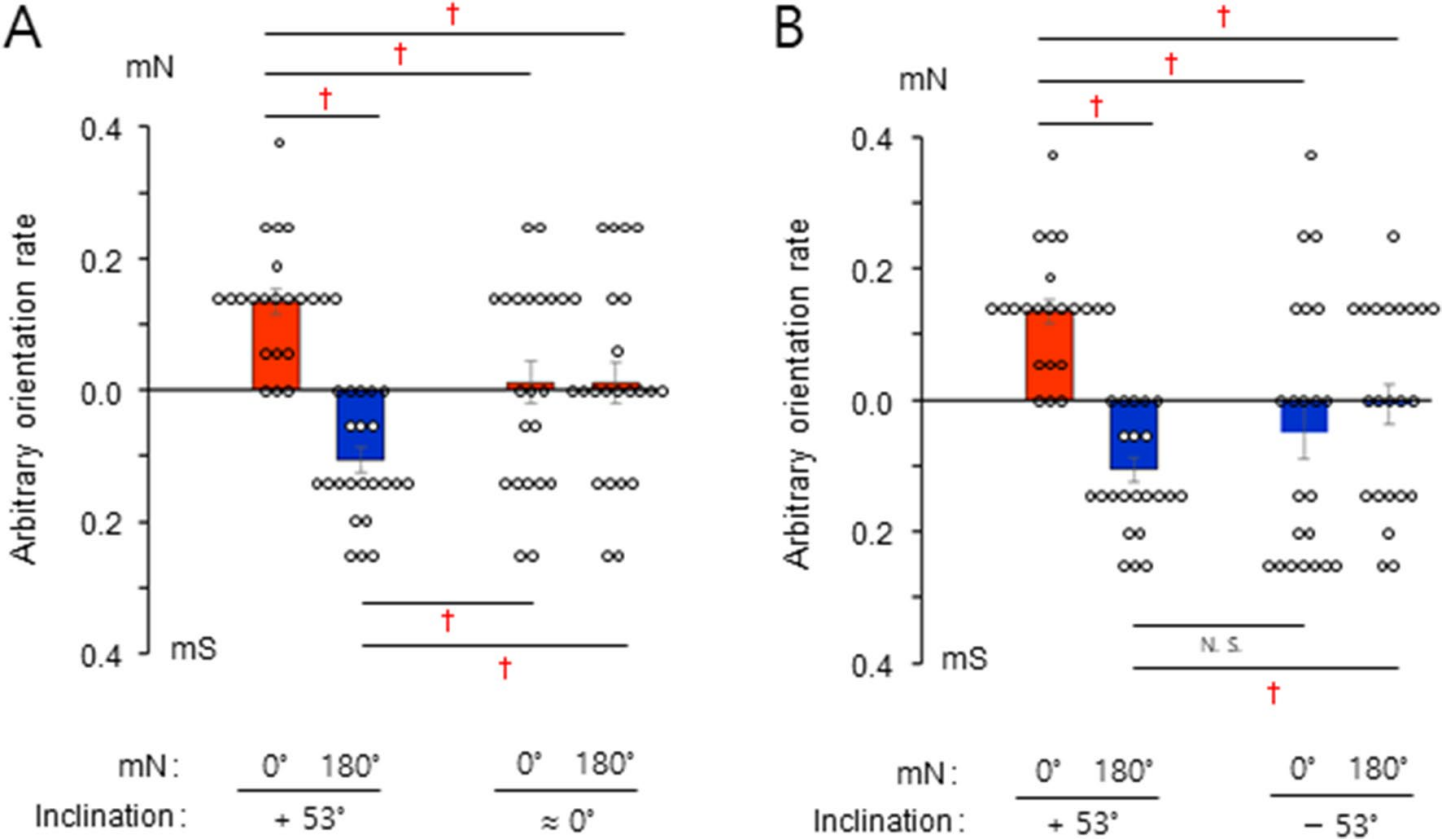
A non-canonical magnetic inclination compass in humans. Selected starved subjects were tested under different inclinations with full wavelength light. The Y axis represents arbitrary orientation rate toward true magnetic north (mN) or true magnetic south (mS). (**A**) Inclination ≈ 0° due to cancellation of the vertical component of the ambient GMF. (**B**) Inclination − 53° due to inversion of the vertical component. The data from the ambient inclination (+ 53°) in (a) and (b) are the same. †, significant; N. S., not significant by a percentile bootstrap analysis (see Appendixes S1 and S2); error bars, standard error of the mean (SEM). Subjects, *n* = 22.

In principle, radical pair-mediated magnetoreception confers an inclination compass^1,2^. The vertical component of the ambient GMF was therefore cancelled in an attempt to learn more about the properties of the magnetic compass^12^. The correct magnetic orientation we had observed for subjects toward true magnetic north (mN) or true magnetic south (mS) when the modulated magnetic north was set to 0° and 180°, respectively, in the control field inclination (+ 53°), was almost eliminated when the inclination was ≈ 0°, indicating that the vertical component was essential and that an inclination compass mediated the observed magnetic orientation (Figs. 4A, S7A,B). Inversion of the vertical GMF component, another diagnostic tool to discriminate between magnetic compass mechanisms^2,12^, produced orientation toward the opposite direction, mS, when the modulated magnetic north was set to 0° (Figs. 4B, S7C). By contrast, an exact reversal of the GMF, i.e., setting magnetic north to 180° and inverting the vertical component, failed to produce significant orientation toward mN or mS, deviating from the notion of a canonical inclination compass (Figs. 4B, S7C). Taken together, the results demonstrate that human magnetic orientation is mediated by a non-canonical inclination compass.

## Discussion

The present study has established the existence of a human magnetic sense to the GMF and for the first time suggested a magnetic field resonance mechanism as the underlying magnetoreception principle. Consistent with findings for some species of birds^35,39^, changes in orientation rates using magnetic fields oscillating at the Larmor frequency, demonstrate that a magnetic field resonance mechanism mediates the human magnetic sense. The effect of an external linearly polarized oscillating magnetic field at the Larmor frequency is known to depend on the angle between the field axis and the ambient GMF^21,35,43^; therefore not only the absence of an effect in the parallel arrangement (0°), but also the enhanced correct orientation rate in the 74° condition provides strong evidence for a probable involvement of radical pair mechanism in humans. Magnetite particles large enough to rotate in the Earth’s magnetic field are not expected to show such effects because they move too slowly to track the direction of a field that reverses more than a million times per second^21^. In agreement with the light-dependent radical pair model and previous studies of birds^24,39^, light effects resulted in distinct orientation profiles with a strong wavelength dependence. Importantly, blue but not green light seems to play a pivotal role in magnetic orientation by overcoming the effect of red light, whereas green light is also sufficient for correct magnetic orientation in birds^25^. The magnetic response to blue light approximately matches the absorption spectrum of fully oxidised FAD in cryptochrome^21,39^ (wavelengths less than 500 nm) and cryptochromes have been reported to occur in the human retina^44^, suggesting that cryptochromes might be promising magnetoreceptor in humans. To understand the complicated food context- and group-dependent orientation profiles, the following is proposed: radical species, e.g., reactive oxygen species such as O_2_^·−^, occur at different concentrations due to different metabolic rates, depending on starvation, body weight or exposed magnetic fields^45–48^. In turn, O_2_^·−^ could form part of a magnetically sensitive cryptochrome-based radical pair in the eyes^35,49–51^ (but see Refs^52,53^). In particular, O_2_^·−^ in the optimal range of concentration may be crucial for significant enhancement of the anisotropic magnetic field effect (direction sensor) through the FADH^·^/O_2_^·−^ radical scavenging system^51^. Besides the evolutionary adaptive scenario^31^, this might be a plausible speculation explaining the superior magnetic sensitivity of men, compared to women with lower basal metabolic rate, under fasting rather than normal feeding. Nevertheless, there might be unknown magnetic sensitivity for women under different contexts, requiring separate investigation. The human inclination compass identified in the present study deviates from the canonical inclination compass, but still maintains the compass function: humans are much less mobile than long-distance migratory birds and may not have evolved to be as sensitive as birds to encountering polarity variation of the GMF. At first sight, the principal results of this study, i.e., blue light and radical pair mechanism-mediated magnetic orientation, are difficult to reconcile with the results of Wang et al.^32^ which suggested a light-independent magnetite-based mechanism for the human magnetic sense. However, the two studies used very different experimental assays that could be probing different receptors. This could be compared to birds, which are thought to have radical pairs in the eyes (for direction sensing) and magnetite elsewhere (for intensity sensing).

The 2-AFC paradigm would be a useful experimental setup particularly for a comparative study on magnetoreception mechanisms, such as radical pair- or magnetite-involved mechanisms. As shown in Fig. S3, the validity and feasibility of the paradigm for the magnetic orientation test are supported by the consistency between the results of the present and previous study^31^ that adopted a rotary chair experiment. The 2-AFC permits rotation within 240° that is larger than the symmetric half of the rotary chair (180°) and subjects can freely rotate clockwise or counterclockwise at any position within the angle in both setups. Therefore, in principle, the difference in rotation extent between the 2-AFC (240°) and the rotary chair (360°) should not necessarily be a difference in the actual magnetic direction search of subjects. In the 2-AFC paradigm, subjects could be more concentrated on the correct magnetic discrimination of one of the two directions rather than precise orientation, for modulated magnetic north on, e.g., the magnetic north–south axis, which was available through the direction referring to the stool that confined the rotation extent. Nonetheless, a significantly correct choice for modulated magnetic north is still a difficult task that would not be accomplished by chance in the absence of real magnetic responsiveness of the subjects as demonstrated in the present study. The 2-AFC paradigm remarkably saved time (≈ 33%) for the experiment on human subjects as described in the Methods section. Given the realistic or potential time limit of subjects impeding human magnetoreception studies in the past and the future, time-saving could be an important advantage. Consequently, researchers may have options to take advantage of a more appropriate test paradigm for their magnetoreception studies on humans.

To gain further insight into the mechanism, future investigations could be undertaken as follows, using the experimental setup of the present study as a platform with or without the oscillating magnetic fields: (1) a magnetic orientation test on subjects with color blindness, including tritanopia, to examine whether functioning photoreceptor cells are necessary for magnetic sensing; (2) identification of magnetoreceptive cells and magnetoreceptors using human eye cultures or eye organoids, combined with spatio-temporal analysis of the magnetoreception process at the molecular level; and (3) electroencephalography cortical mapping of the magnetic sense area to examine whether the radical pair mechanism is critical under different lights or food contexts.

## Methods

### Subjects

The study comprised 34 men (19–26 years, mean 23 years; body mass index 19–31 kg/m^2^, mean 24 kg/m^2^) with no physical disabilities or mental disorders, including color blindness and claustrophobia^30,31^. All subjects were informed of the aims, the study procedure, and the financial compensation for participation, and were asked to follow the rules of the study. Prior to each experiment, subjects underwent short-term starvation^31,54^ (18–20 h; no food except pure water after lunch (12–1 pm) or dinner (6–7 pm), no later than 1 pm or 7 pm, respectively, one the day before the test), no medical treatments, and normal sleep (at least 6 h, between 10 pm the day before the test day to 8 am on the test day)^31^. Prior to starting each experiment, subjects were stabilized on a chair for ~ 10 min in a room next to the testing room. Based on an assessment with a pre-experiment questionnaire and the first blood glucose level, measured before starting the experiment (see “Geomagnetic orientation assay” section below), subjects who had not followed these rules were not allowed to take the test on the day and the test was postponed. The study was approved by the Institutional Review Board of Kyungpook National University and all the procedures followed the regulations for human subject research. Informed consent was obtained from all subjects.

### Modulation of GMF

The ambient GMF in the testing room had a total intensity 45.0 μT, inclination 53°, and declination − 7° (Daegu city, Republic of Korea); the total intensity of 50.0 μT in our previous study^31^ was changed due to a reconstruction of the building; 45.0 μT was maintained throughout the period of this study. To provide the subjects with various GMF-like magnetic fields (i.e., by modulating of total intensity, inclination, or direction of magnetic north), the coil system from our previous studies^6,7,31^ was used. It comprised three double-wrapped, orthogonal, rectangular Helmholtz coils (1.890 × 1.890 m, 1.890 × 1.800 m, and 1.980 × 1.980 m for the north–south, east–west, and vertical axes, respectively), electrically-grounded with copper mesh shielding. The subject was seated on a rotatable plastic chair with no metal components, at the center of the three-dimensional coils with his head positioned in the middle space of the vertical axis of the coils. To modulate the geomagnetic north, each pair of coils was supplied with direct current from a power supply (MK3003P; MKPOWER, Republic of Korea). The magnetic field was measured using a 3-axis magnetometer (MGM 3AXIS; ALPHALAB, USA); the field homogeneity at the position of the subject’s head was found to be 95%. The testing room was shielded by a six-sided Faraday cage comprising 10 mm thick aluminum plates, and was grounded during the entire experiment^40^. Background electromagnetic noise was measured inside the coils at the start and the end of each experimental day. It was attenuated by the Faraday cage more than 200-fold over the range from 500 Hz to 100 MHz as described in detail in our previous study^31^. The 60 Hz power frequency magnetic field was no more than 2 nT (3D NF Analyzer NFA 1000; Gigahertz Solutions, Germany). All electronic devices were placed outside the Faraday cage during the experiments, with the exception of the switch button module for GMF modulation and the antenna for generating the oscillating magnetic fields. The temperature experienced by the subjects was maintained at 25 ± 0.5 °C (Data logger 98,581; MIC Meter Industrial, Taiwan) by air circulation through the honeycomb on the ceiling of the Faraday cage^31^.

### Geomagnetic orientation assay

Adopting a two-alternative forced choice (2-AFC) paradigm^33,34^, a geomagnetic orientation assay was conducted similar to our previous study^31^. Experiments were performed at 09:30–11:30 am or 1:00–5:00 pm (local time, UTC + 09:00) (each experiment: 50 min–1 h 10 min; mean ≈ 1 h, which was shorter by approximately 30 min than that in the previous study: 1 h 20 min–1 h 40 min; mean ≈ 1 h 30 min). Depending on the experiment, starved or unstarved subjects were tested individually. Prior to each experiment, the subjects were asked to remain with their heads facing the front, with eyes closed and earmuffs on during the experiment. In particular, they were asked to concentrate on sensing, if they could, the ambient geomagnetic north during the association phase, and to use the sensed information, depending on the experiment, to orient toward one of the two modulated magnetic norths (0°/180° for magnetic north–south axis or 90°/270° for magnetic east–west axis, rotated clockwise with respect to the ambient geomagnetic north) during the test phase. Subjects were instructed to avoid distracting thoughts and to think immediately “which direction is modulated magnetic north?” whenever they were distracted during the test phase, or felt they were being biased by experiences from earlier experiments. While seated on the rotatable chair, the subject’s blood glucose level was measured shortly before the first session and immediately after each session with eyes open except in the ‘dark’ experiment (Accu-Chek Guide; Roche, Germany)^31^. If the determined value before the first session varied by more than 15% relative to the mean (Table S2)^31^, the experiment was postponed and repeated at a later date (approximately 2% of experiments). The subjects were stabilized with eyes closed for 2 min before the first trial in the absence of visual, auditory, olfactory, and haptic sensory cues. For the ‘dark’ experiment (light intensity ≈ 0 lx), subjects wore home-made ‘blind’ goggles and were stabilized with eyes closed for 5 min^55,56^, and then asked whether they could see any light. If they could, the goggles were adjusted to prevent leakage of light, and the subject then had another 5 min of stabilization with eyes closed before starting the experiment. The subjects were illuminated with light from a filtered/non-filtered diffused light-emitting diode, depending on the experiment (Table S1). The home-made filter goggles were worn throughout the experiment, including the association and test phase, when required. The goggles contained glass filters (Tae Young Optics, Republic of Korea) to provide the eyes with particular wavelengths of light (Spectrometer USB4000-UV-VIS, Ocean Optics, USA) (Fig. S1). Each experiment consisted of 16 sequential trials for ‘no-association’ and ‘food-association’. For the food-association, a subject facing toward the ambient geomagnetic north was gently provided with a chocolate chip^31^ on his right palm by an experimenter, and given 30 s to eat it, while during no-association trials, food was not provided during the association phase. After a subsequent 5 s interval, the experimenter gently touched the subject’s right thenar area using a paper rod, as the cue to start the test. One of the two modulated magnetic north directions, depending on the experiment, was randomly provided 3 s before the cue for the test. Each of the modulated magnetic north directions was provided eight times for the no-association and food-association sessions. Subjects were informed of the nearly equal probability for each of the modulated magnetic north directions before each experiment. With the touch cue, subjects were asked to rotate freely toward any direction (clockwise or counterclockwise) by themselves (1–4 cycles of two-thirds rotation) and try to sense the direction of the modulated magnetic north during a 1 min period. Rotation was allowed within the rotation angle (− 30° to 210° for the magnetic north–south axis or − 120° to 120° for the east–west axis, depending on experiments, with respect to the ambient magnetic north), which was confined by the plastic stool (Fig. 1A) touching the left or right ankle of the subjects. When subjects determined the direction of the magnetic north, they stopped rotating to face toward the direction and lifted their right hand to indicate the direction to the experimenter. The direction was measured by the experimenter at 10° intervals using the scale on the walls of the Faraday cage^31^. A prerequisite for correct orientation was that the subject indicated the direction within the range of 30° to the both sides with respect to the magnetic cardinal directions, which was instructed to the subjects before each experiment. When the direction the subjects indicated was out of the 30° range, the trial was not included in the data and was repeated (approximately 0.63% of trials). Before the subsequent trial, the subject was gently rotated to face toward the ambient geomagnetic north and then rested for 5 s. For the ‘dark’ experiment, subjects were asked whether they could see any leaked light immediately after the last measurement of blood glucose level at the end of experiment. If the subject could see leaked light, the experiment was nullified and repeated later on (approximately 3% of experiments; 2/68). All experiments were performed in a double-blind fashion. The experimenter who conducted the orientation assay knew whether a subject was starved or not, wearing filter goggles, and food-associated or not, but did not know the random magnetic north sequences that were controlled by the personal computer (PC) system. Another experimenter responsible for analyzing the data did not know whether the subject was starved or not, the experimental conditions, including light wavelengths, or whether an oscillating magnetic field had been provided to the subjects. Thus, none of the experimenters were aware of all the subject information and data during the experiments and data analysis. The correct orientation rate was calculated by (the number of correct orientation trials/total number of trials) (raw data, Appendix S3). All the subjects participated in all the experiments performed in random order with an interval of at least 3 days between experiments. After each experiment, the subjects were asked to answer a post-experiment questionnaire about whether they closed their eyes when required during the entire period of the experiment. In cases when a subject did not maintain closed eyes, the experiment was repeated (approximately 1% of experiments). For each subject, a preliminary experiment on the “magnetic north–south axis” was conducted twice (unstarved and starved for each) with no goggles for adaption to the experimental procedure. These data were not included in the results.

### Experiments with oscillating magnetic fields

Experiments with oscillating magnetic fields were performed using the standard geomagnetic orientation assay described above. To produce the oscillating magnetic fields, oscillating currents from a function generator (AFG3021; Tektronix, USA. For each magnetic field, sweep of 500 ms; interval of 1 ms. See Fig. S6A) were amplified (ENI 2100L RF power amplifier; Bell Electronics, USA) and fed into a calibrated coil antenna (30 cm diameter, 6509 loop antenna; ETS-LINDGREN, USA) mounted on a wooden frame, comprised of a single winding of coaxial cable. The oscillating magnetic fields were measured daily, before the first and after the last experiment of the day, using a spectrum analyzer (SPA-921TG; Com-Power, USA) with a calibrated loop antenna (48 cm diameter, AL-130R; Com-Power, USA) and a calibrated magnetometer (Probe HF 3061, NBM-550; Narda, Germany). Magnetic field intensities were measured on the glabella of the subjects; variations in intensity between subjects due to different seating heights were less than 10% of the average values (Table S3). The function generator and amplifier were placed outside the Faraday cage, and switched on during the dummy load control experiments with no signal from the PC system. The band widths of the monochromatic magnetic fields, i.e., 1.260 and 1.890 MHz were 0.020 and 0.019 MHz (“average”, √3 kHz), respectively, at the bottoms of the peaks. During the test phase, the maximum values of magnetic noise on the glabella of subjects including the dummy load did not exceed the following values: (1) 5 Hz–9 kHz; 2 nT/√ 2 kHz of “average” and 8 nT/√ 9 kHz of “max-hold” (0.05 nT/√ 2 kHz of “average” and 5 nT/√ 9 kHz of “max-hold” in the dummy load) (3D NF Analyzer NFA 1000; Gigahertz Solutions, Germany); (2) 9 kHz–500 kHz; 5 nT/√ 3 kHz of “average” and 8 nT/√ 3 kHz of “max-hold” (≈ 0 nT/√ 3 kHz of “average” and ≈ 1 nT/√ 3 kHz of “max-hold” in the dummy load) (the AL-130R antenna) (Fig. S6C); and (3) 500 kHz–30 MHz; 0.006 nT of 3.780 MHz harmonic in the 1.260 MHz, 0.03 nT of 5.670 MHz harmonic in the 1.890 MHz, and ≈ 0 nT in the dummy load (/√ 10 kHz of “average”) (Fig. S6B), and 0.15 nT/√ 10 kHz of “max-hold” at the same frequencies above and ≈ 0 nT in the dummy load (the AL-130R antenna).

### Statistical analysis

To determine the significance of orientation data from the 2-AFC paradigm, a one-sample *t*-test (test mean: 0.5), paired sample *t*-test, or two-sample *t*-test was performed using Origin software 11 (Origin, USA). To verify the reasonability of the *t*-tests, all data sets were checked using the Anderson–Darling test if the data follow a normal distribution (Appendix S4). To evaluate the difference between the means of two data sets when at least one of them did not show a normal distribution, the percentile bootstrap method^57^ was used (95% confidence interval, see Fig. S2, Appendices S1 and S2 for raw data). To analyze the blood glucose data, a paired sample *t*-test was used. Based on the results of previous study^31^, to describe different response groups of magnetic orientation in the 2-AFC paradigm, a principal component analysis^36,37^ was conducted on correct orientation rates by starved subjects, with no association/food-association under the full wavelength or > 400 nm light conditions using SPSS 23 (IBM, USA). Following the principal component analysis calculation, the *k*-means clustering algorithm—one of the unsupervised learning methods—was used to objectively classify the groups^58^. The number of groups was two, and the distance between the center of the cluster and all points was Euclidean distance. The classification boundary was marked with the perpendicular bisector from the centers of the two groups. The first two principal components accounted for a significant portion of the total variance (73.1%; PC1 = 40.8%, PC2 = 32.3%). Statistical values are presented as mean ± SEM.

## Supplementary Information

Supplementary Information 1.

Supplementary Information 2.

Supplementary Information 3.

Supplementary Information 4.

Supplementary Figures and Tables.

## References

[1] Johnsen S, Lohmann KJ (2005). The physics and neurobiology of magnetoreception. Nat. Rev. Neurosci..

[2] Wiltschko R, Wiltschko W (2006). Magnetoreception. BioEssays.

[3] Lohmann KJ, Putman NF, Lohmann CM (2012). The magnetic map of hatchling loggerhead sea turtles. Curr. Opin. Neurobiol..

[4] Guerra PA, Gegear RJ, Reppert SM (2014). A magnetic compass aids monarch butterfly migration. Nat. Commun..

[5] Gegear R, Casselman A, Waddell S, Reppert SM (2008). Cryptochrome mediates light-dependent magnetosensitivity in *Drosophila*. Nature.

[6] Bae JE (2016). Positive geotactic behaviors induced by geomagnetic field in *Drosophila*. Mol. Brain.

[7] Oh IT (2020). Behavioral evidence for geomagnetic imprinting and transgenerational inheritance in fruit flies. Proc. Natl. Acad. Sci. USA.

[8] Begall S, Cerveny J, Neef J, Vojtech O, Burda H (2008). Magnetic alignment in grazing and resting cattle and deer. Proc. Natl. Acad. Sci. USA.

[9] Hart V (2013). Dogs are sensitive to small variations of the Earth’s magnetic field. Front. Zool..

[10] Bazalova O (2016). Cryptochrome 2 mediates directional magnetoreception in cockroaches. Proc. Natl. Acad. Sci. USA.

[11] Maffei ME (2014). Magnetic field effects on plant growth, development, and evolution. Front. Plant Sci..

[12] Wiltschko W, Wiltschko R (1972). Magnetic compass of European robins. Science.

[13] Boles LC, Lohmann KJ (2003). True navigation and magnetic maps in spiny lobsters. Nature.

[14] Blakemore R (1975). Magnetotactic bacteria. Science.

[15] Phillips JB, Borland SC (1992). Behavioural evidence for use of a light-dependent magnetoreception mechanism by a vertebrate. Nature.

[16] Phillips JB (1986). Two magnetoreception pathways in a migratory salamander. Science.

[17] Marhold S, Wiltschko W, Burda H (1997). A magnetic polarity compass for direction finding in a subterranean mammal. Naturwissenschaften.

[18] Lohmann K (1995). Magnetic orientation of spiny lobsters in the ocean: Experiments with undersea coil systems. J. Exp. Biol..

[19] Schulten K, Swenberg CE, Weiler A (1978). A biomagnetic sensory mechanism based on magnetic field modulated coherent electron spin motion. Z. Phys. Chem..

[20] Ritz T, Adem S, Schulten K (2000). A model for photoreceptor-based magnetoreception in birds. Biophys. J..

[21] Hore PJ, Mouritsen H (2016). The radical-pair mechanism of magnetoreception. Annu. Rev. Biophys..

[22] Mann S, Sparks NH, Walker MM, Kirschvink JL (1988). Ultrastructure, morphology and organization of biogenic magnetite from sockeye salmon, *Oncorhynchus nerka*: Implications for magnetoreception. J. Exp. Biol..

[23] Xu J (2021). Magnetic sensitivity of cryptochrome 4 from a migratory songbird. Nature.

[24] Wiltschko R, Stapput K, Thalau P, Wiltschko W (2010). Directional orientation of birds by the magnetic field under different light conditions. J. R. Soc. Interface.

[25] Wiltschko W, Munro U, Ford H, Wiltschko R (1993). Red light disrupts magnetic orientation of migratory birds. Nature.

[26] Maslanyj M, Lightfoot T, Schüz J, Sienkiewicz Z, McKinlay A (2010). A precautionary public health protection strategy for the possible risk of childhood leukaemia from exposure to power frequency magnetic fields. BMC Public Health.

[27] Vadalà M (2016). Mechanisms and therapeutic effectiveness of pulsed electromagnetic field therapy in oncology. Cancer Med..

[28] Baker RR (1980). Goal orientation by blindfolded humans after long-distance displacement: Possible involvement of a magnetic sense. Science.

[29] Westby GW, Partridge KJ (1986). Human homing: Still no evidence despite geomagnetic controls. J. Exp. Biol..

[30] Mulligan BP, Persinger MA (2012). Experimental simulation of the effects of sudden increases in geomagnetic activity upon quantitative measures of human brain activity: Validation of correlational studies. Neurosci. Lett..

[31] Chae KS, Oh IT, Lee SH, Kim SC (2019). Blue light-dependent human magnetoreception in geomagnetic food orientation. PLoS ONE.

[32] Wang, C. X. *et al.* Transduction of the geomagnetic field as evidenced from alpha-band activity in the human brain. *eNeuro*. **6**, ENEURO.0483-18.2019. 10.1523/ENEURO.0483-18.2019 (2019). 10.1523/ENEURO.0483-18.2019 PMC6494972 31028046

[33] Azzopardi P, Cowey A (1997). Is blindsight like normal, near-threshold vision?. Proc. Natl. Acad. Sci. USA.

[34] Tinsley JN (2016). Direct detection of a single photon by humans. Nat. Commun..

[35] Ritz T, Thalau P, Phillips JB, Wiltschko R, Wiltschko W (2004). Resonance effects indicate a radical-pair mechanism for avian magnetic compass. Nature.

[36] Jolliffe IT, Cadima J (2016). Principal component analysis: A review and recent developments. Philos. Trans. A. Math. Phys. Eng. Sci..

[37] Mure LS, Vinberg F, Hanneken A, Panda S (2019). Functional diversity of human intrinsically photosensitive retinal ganglion cells. Science.

[38] Phillips JB, Jorge PE, Muheim R (2010). Light-dependent magnetic compass orientation in amphibians and insects: Candidate receptors and candidate molecular mechanisms. J. R. Soc. Interface..

[39] Wiltschko R, Wiltschko W (2014). Sensing magnetic directions in birds: Radical pair processes involving cryptochrome. Biosensors (Basel)..

[40] Engels S (2014). Anthropogenic electromagnetic noise disrupts magnetic compass orientation in a migratory bird. Nature.

[41] Schwarze S (2016). Weak broadband electromagnetic fields are more disruptive to magnetic compass orientation in a night-migratory songbird (*Erithacus rubecula*) than strong narrow-band fields. Front. Behav. Neurosci..

[42] Januszkiewicz Ł (2018). Analysis of human body shadowing effect on wireless sensor networks operating in the 2.4 GHz band. Sensors (Basel).

[43] Thorsten R (2009). Magnetic compass of birds is based on a molecule with optimal directional sensitivity. Biophys. J..

[44] Thompson CL (2003). Expression of the blue-light receptor cryptochrome in the human retina. Invest. Ophthalmol. Vis. Sci..

[45] Chen Y, Azad MB, Gibson SB (2009). Superoxide is the major reactive oxygen species regulating autophagy. Cell Death Differ..

[46] Wohaieb SA, Godin DV (1987). Starvation-related alterations in free radical tissue defense mechanisms in rats. Diabetes.

[47] Arthaut L-D (2017). Blue-light induced accumulation of reactive oxygen species is a consequence of the *Drosophila* cryptochrome photocycle. PLoS ONE.

[48] Sherrard RM (2018). Low-intensity electromagnetic fields induce human cryptochrome to modulate intracellular reactive oxygen species. PLoS Biol..

[49] Behndig A, Svensson B, Marklund SL, Karlsson K (1998). Superoxide dismutase isoenzymes in the human eye. Invest. Ophthalmol. Vis. Sci..

[50] Solov'yov IA, Schulten K (2009). Magnetoreception through cryptochrome may involve superoxide. Biophys. J..

[51] Kattng DR (2017). Radical-pair-based magnetoreception amplified by radical scavenging: Resilience to spin relaxation. J. Phys. Chem. B..

[52] Hogben HJ, Efimova O, Wagner-Rundell N, Timmel CR, Hore PJ (2009). Possible involvement of superoxide and dioxygen with cryptochrome in avian magnetoreception: Origin of Zeeman resonances observed by *in vivo* EPR spectroscopy. Chem. Phys. Lett..

[53] Player TC, Hore PJ (2019). Viability of superoxide-containing radical pairs as magnetoreceptors. J. Chem. Phys..

[54] Romijn JA, Godfried MH, Hommes MJ, Endert E, Sauerwein HP (1990). Decreased glucose oxidation during short-term starvation. Metabolism.

[55] Ramamurthy M, Lakshminarayanan V (2015). Human Vision and Perception. Handbook of Advanced Lighting Technology.

[56] Ando K, Kripke DF (1996). Light attenuation by the human eyelid. Biol. Psychiatry.

[57] Efron, B. The jackknife, the bootstrap and other resampling plans. In *CBMS-NSF Regional Conference Series in Applied Mathematics, Monograph*, vol. 38 (SIAM, 1982).

[58] Lloyd SP (1982). Least squares quantization in PCM. IEEE Trans. Inf. Theory..

