# Supplementary material for: Human magnetic sense is mediated by a light and magnetic field resonance-dependent mechanism
Source: Sci Rep. 2022 May 30;12:8997. doi: 10.1038/s41598-022-12460-6 (PMC9151822; doi:10.1038/s41598-022-12460-6)
Supplement: Supplementary file 5 — Supplementary Figures and Tables. [file 41598_2022_12460_MOESM5_ESM.pdf]

Supplementary Information

Human magnetic sense is mediated by a light and magnetic field resonance-dependent mechanism

Kwon-Seok Chae[1,2,3,6,✉], Soo-Chan Kim[4,6], Hye-Jin Kwon[2], Yongkuk Kim[5]

[1]Department of Biology Education, Kyungpook National University; Daegu 41566, Republic of Korea. [2]Department of Nanoscience & Nanotechnology, Kyungpook National University; Daegu 41566, Republic of Korea. [3]Brain Science and Engineering Institute, Kyungpook National University; Daegu 41566, Republic of Korea.[4]Department of Electrical and Electronic Engineering, Research Center for Applied Human Sciences, Hankyong National University; Anseong 17579, Republic of Korea. [5] Department of Mathematics, Kyungpook National University, Daegu 41566, Republic of Korea. [6] These authors contributed equally: Kwon-Seok Chae and Soo-Chan Kim. ✉ email: kschae@knu.ac.kr

Correspondence and requests for materials should be addressed to K.-S.C.

This PDF file includes:

Figs. S1 to S7
Tables S1 to S3

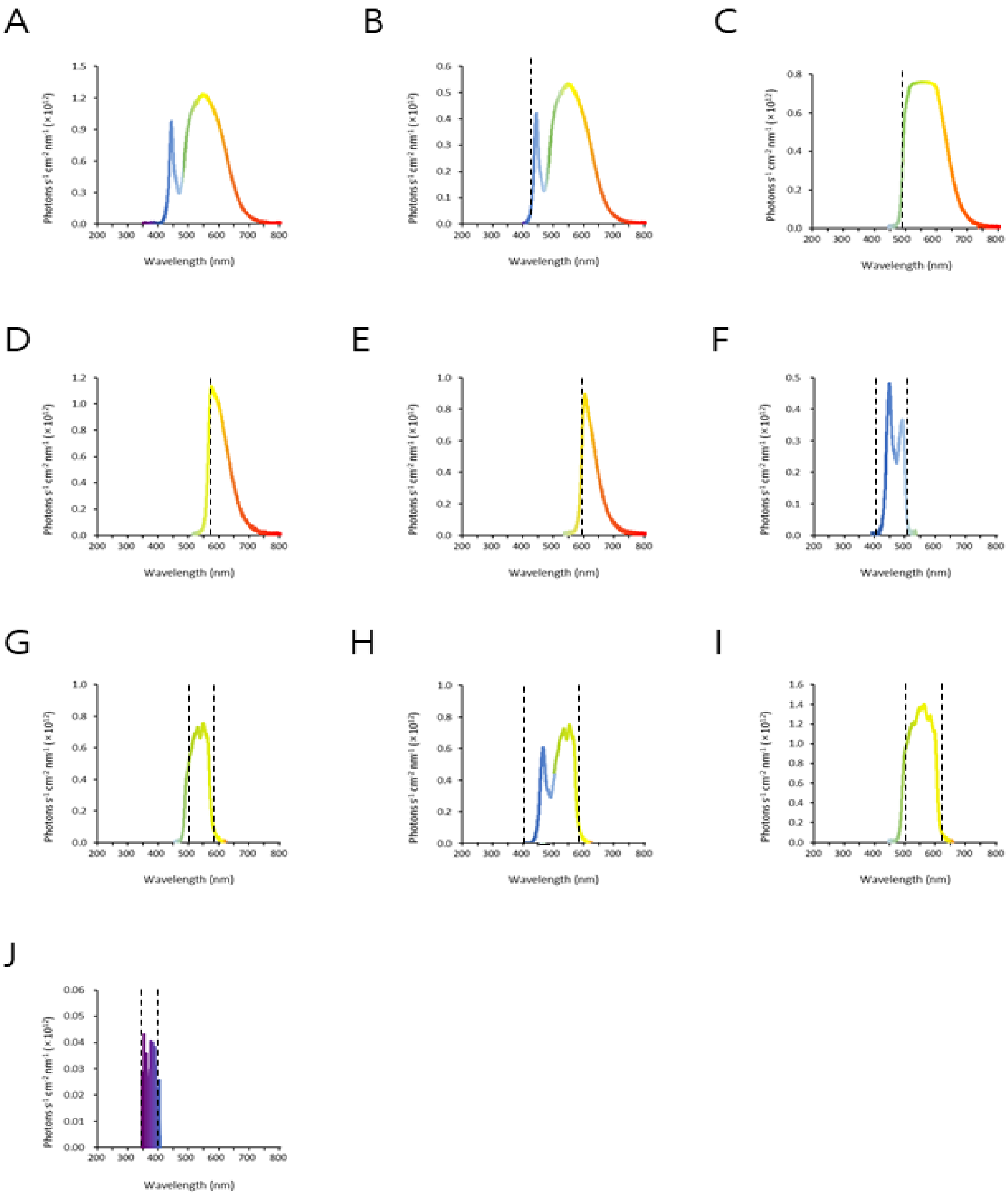
A
B
C
D
E
F
G
H
I
J
Wavelength (nm)
Photons s-1 cm-2 nm-1 (×10^12)

Fig. S1. Spectra of the lights used in the experiments.

(**A**)–(**J**) Spectra of light-emitting diodes without goggles (350–800 nm) (A) or filtered by one of the filter goggles: 400–800 nm (B), 500–800 nm (C), 570–800 nm (D), 600–800 nm (E), 400–500 nm (F), 500–570 nm (G), 400–570 nm (H), 500–600 nm (I), and 350–400 nm (J). Dashed lines denote the cutoff points of the filters by light transmittance of > 50%. Each spectrum is the mean of two repeated measurements.

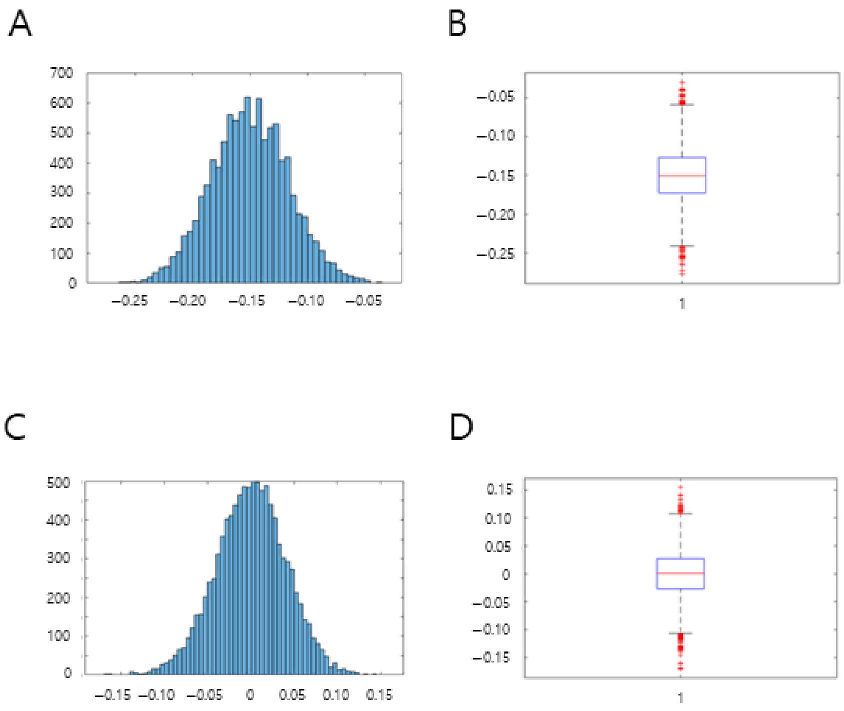
A
B
C
D
700
600
500
400
300
200
100
0
−0.25 −0.20 −0.15 −0.10 −0.05
−0.05
−0.10
−0.15
−0.20
−0.25
1
500
400
300
200
100
0
−0.15 −0.10 −0.05 0 0.05 0.10 0.15
0.15
0.10
0.05
0
−0.05
−0.10
−0.15
1

Fig. S2. Percentile bootstrap analysis to test statistical differences in Fig. 1B.

(A) and (B) Histogram and boxplot of percentile bootstrap analysis for the data of no-association and food-association in Group 1. **(C) and (D)** Histogram and boxplot of percentile bootstrap analysis for the data of no-association in Group 1 and Group 2. Details, see Appendixes S1, S2, and S4.

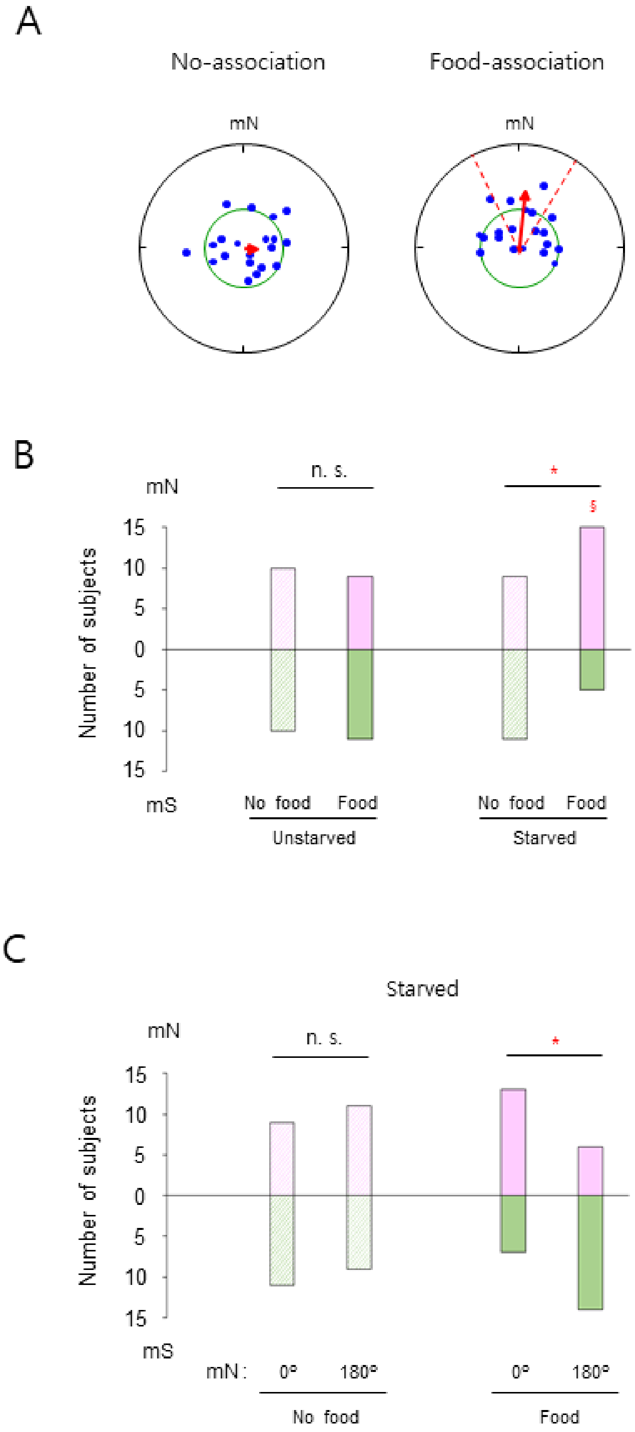
A
No-association
Food-association
mN
mN
B
mN
n. s.
*
§
Number of subjects
15
10
5
0
5
10
15
mS
No food
Food
No food
Food
Unstarved
Starved
C
Starved
mN
n. s.
*
Number of subjects
15
10
5
0
5
10
15
mS
mN :
0°
180°
0°
180°
No food
Food

Fig. S3. A reanalysis of Fig. 1B-related data in our previous study.

(**A**) Reanalyzed data were from Fig. 2C, D in Chae et al.[31]. Circular diagrams of geomagnetic orientation of the starved male subjects. The reanalyzed data were from trials in which the modulated magnetic north was randomly set at 0° or 180°, i.e., on the ambient magnetic north-south axis. Orientation toward modulated magnetic north was significant in the food-association case only. (*Left*) No-association. $\alpha = 96.8°$, $r = 0.13$, $P = 0.54$ (V test), $P = 0.73$ (Rayleigh test), $n = 20$. (*Right*) Food-association. $\alpha = 7.4°$, $r = 0.54$, $P = 0.00023$ (V test; 95% confidence interval; 337.3°–37.4°), $P = 0.002$ (Rayleigh test), $n = 20$. The V test was more appropriate to evaluate whether the subjects oriented toward the magnetic north. In each circular diagram, the dots and arrow indicate the subject's mean direction vector and the group mean vector, respectively. The solid circle and dashed lines indicate the minimum length of the group mean vector and the confidence interval needed for significance in the V test ($P = 0.05$), respectively. mN, the modulated magnetic north; α, group mean vector as clockwise angle with respect to modulated magnetic north; r, length of group mean vector. (**B**) The reanalyzed data were from Fig. 2A, C, D, and Fig. S2A in Chae et al.[31]. Note that the Y axis represents number of subjects who oriented toward magnetic north (mN) or magnetic south (mS) with respect to the modulated magnetic north. Each subject's orientation toward mN or mS was determined by whether his mean direction vector (μ) belonged to $270.0° < \mu < 90.0°$ (mN) or $90.0° < \mu < 270.0°$ (mS) by clockwise rotation with respect to modulated magnetic north. The number of subjects oriented toward mN was only significant in the food-association of starved subjects. §, $P < 0.05$ by one-sample proportion test; *, $P < 0.05$ by two-sample proportion test. (**C**) The data in starved

condition of (B) above were reanalyzed. Note that the Y axis represents the number of subjects who oriented toward true magnetic north (mN) or true magnetic south (mS). In this case, each subject's orientation toward mN or mS was determined by whether his mean direction vector (μ) belonged to 270.0° < μ < 90.0° (mN) or 90.0° < μ < 270.0° (mS) by clockwise rotation with respect to true magnetic north. The significance in the food-association case was due to the remarkable correct orientations toward both 0° and 180°, which were the directions modulated magnetic north was set on during the test phase. *, $P < 0.05$ by two-sample proportion test. In this figure, the number of subjects was $n = 20$.

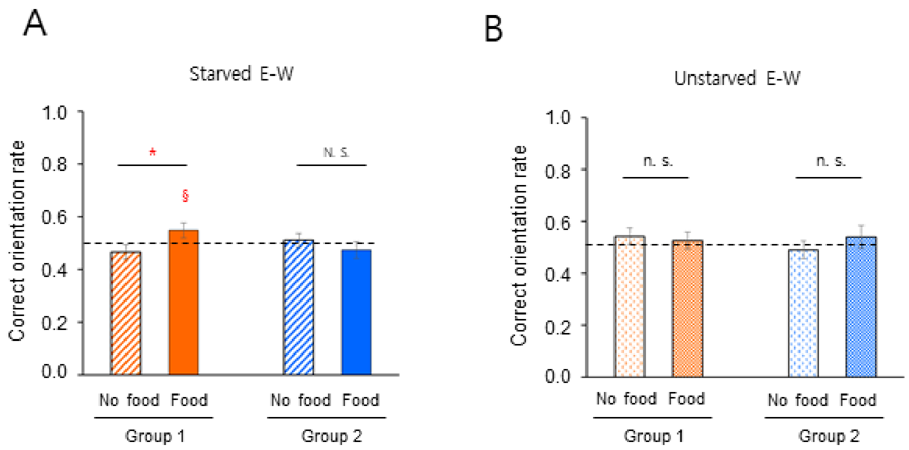
A
Starved E-W
Correct orientation rate
1.0
0.8
0.6
0.4
0.2
0.0
*
§
N. S.
No food
Food
No food
Food
Group 1
Group 2
B
Unstarved E-W
Correct orientation rate
1.0
0.8
0.6
0.4
0.2
0.0
n. s.
n. s.
No food
Food
No food
Food
Group 1
Group 2

Fig. S4. Magnetic orientation on the magnetic east-west axis.

The modulated magnetic north was randomly set to either 90° or 270°, instead of 0° or 180°. (**A**) A significant increase (Group 1) and marginal decrease (Group 2) in correct orientation rate between the different associations under the starved condition. §, $P < 0.05$ by one-sample *t*-test; *, $P < 0.05$ by paired sample *t*-test. N. S., not significant by a percentile bootstrap analysis (see Appendixes S1 and S2) (**B**) No notable change of correct orientation rate under the unstarved condition. n.s., not significant by paired sample *t*-test. Horizontal dashed lines, 0.5 for correct orientation rate. Group 1, $n = 20$ and Group 2, $n = 14$.

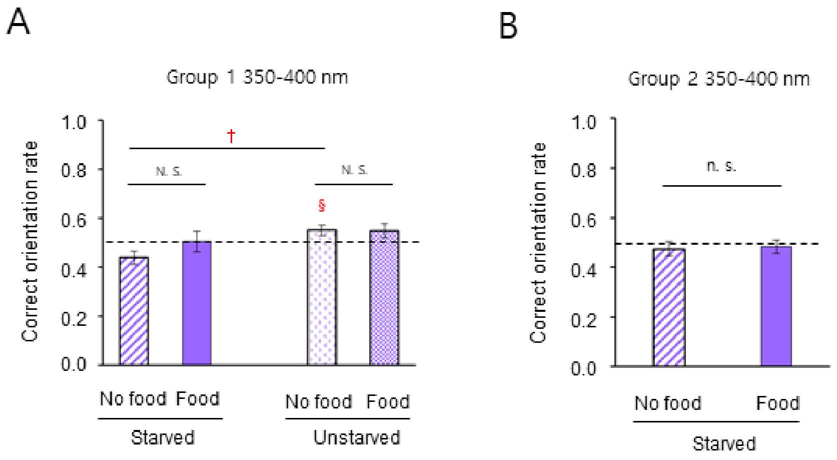
A
Group 1 350-400 nm
Correct orientation rate
1.0
0.8
0.6
0.4
0.2
0.0
†
N. S.
N. S.
§
No food Food
Starved
No food Food
Unstarved
B
Group 2 350-400 nm
Correct orientation rate
1.0
0.8
0.6
0.4
0.2
0.0
n. s.
No food
Food
Starved

Fig. S5. Magnetic orientation with UV-A light by subject group.

Correct magnetic orientation rate with 350–400 nm light in Group 1 starved or unstarved (**A**) and Group 2 starved (**B**). †, significant; N. S., not significant by a percentile bootstrap analysis (see Appendixes S1 and S2); §, $P < 0.05$ by one-sample *t*-test; n.s., not significant by paired sample *t*-test; horizontal dashed lines, 0.5 for correct orientation rate; error bars, standard error of the mean (SEM). Group 1, $n = 20$ and Group 2, $n = 14$.

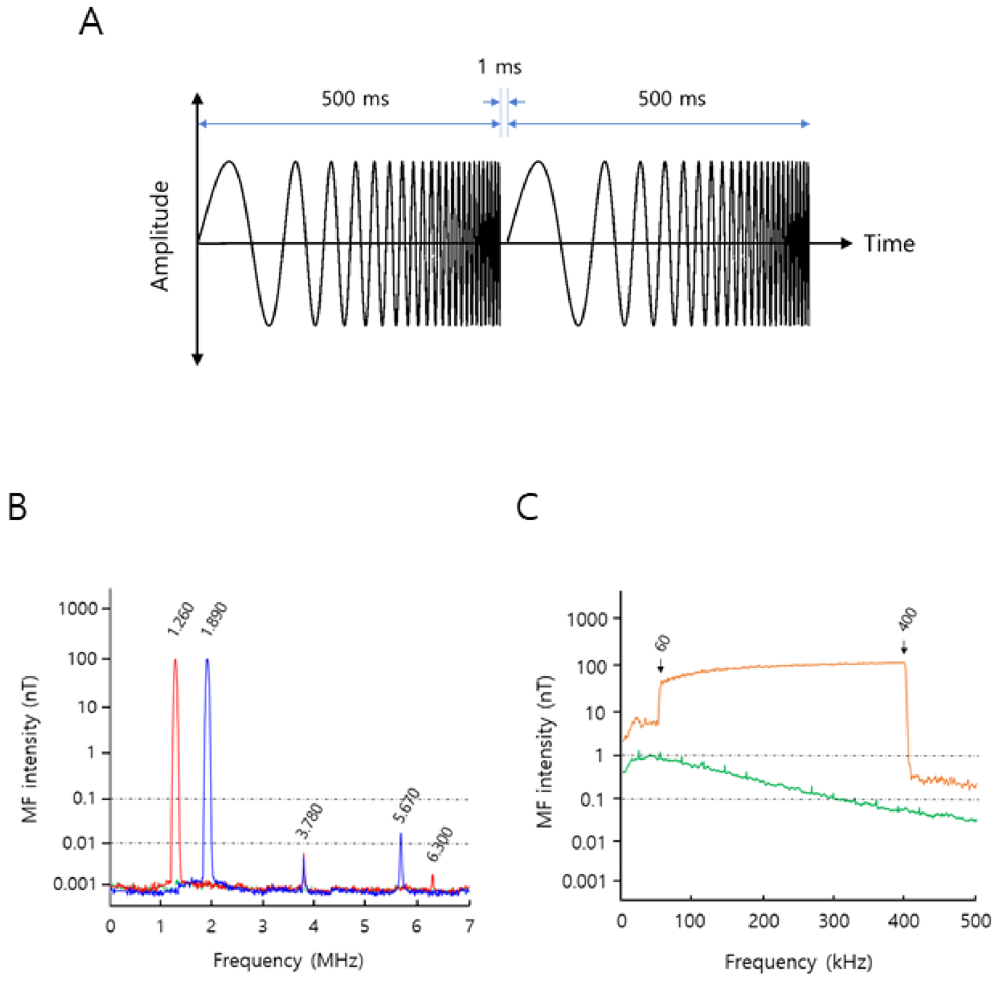
A
1 ms
500 ms
500 ms
Amplitude
Time
B
MF intensity (nT)
1000
100
10
1
0.1
0.01
0.001
1.260
1.890
3.780
5.670
6.300
0 1 2 3 4 5 6 7
Frequency (MHz)
C
MF intensity (nT)
1000
100
10
1
0.1
0.01
0.001
60
400
0 100 200 300 400 500
Frequency (kHz)

Fig. S6. Magnetic field conditions used in the oscillatory magnetic field experiments. (**A**) The waveform of the 0.060–0.400 MHz magnetic field as a representative waveform of the oscillating magnetic fields. Each of the oscillating magnetic fields, i.e., 1.260, 1.890, and 0.060–0.400 MHz was generated using the function generator (sweep, 500 ms; interval, 1 ms). (**B**) and (**C**) Magnetic field measurements of test conditions in the 1.260 and 1.890 MHz (B) and 0.060–0.400 MHz (C) magnetic field experiments, respectively. The magnetic intensity was "average" (√10 kHz) in (B) and "max-hold" (√10 kHz) in (C). Each of the values over the peaks indicates the center frequency of the corresponding peak. Note the lower levels of magnetic noise in the dummy loads, and harmonic frequencies; 3.780 and 6.300 of the 1.260 MHz, and 3.780 and 5.670 of the 1.890 MHz magnetic fields in (B), and between 0 and 60 Hz, and > 400 kHz in (C). For measurement details, including the sharpness of peaks, see the Methods. Different colors of the lines indicate the signals of 1.260 (red), 1.890 (blue), and 0.060–0.400 (orange) MHz magnetic fields, and the magnetic noises of the dummy loads (green).

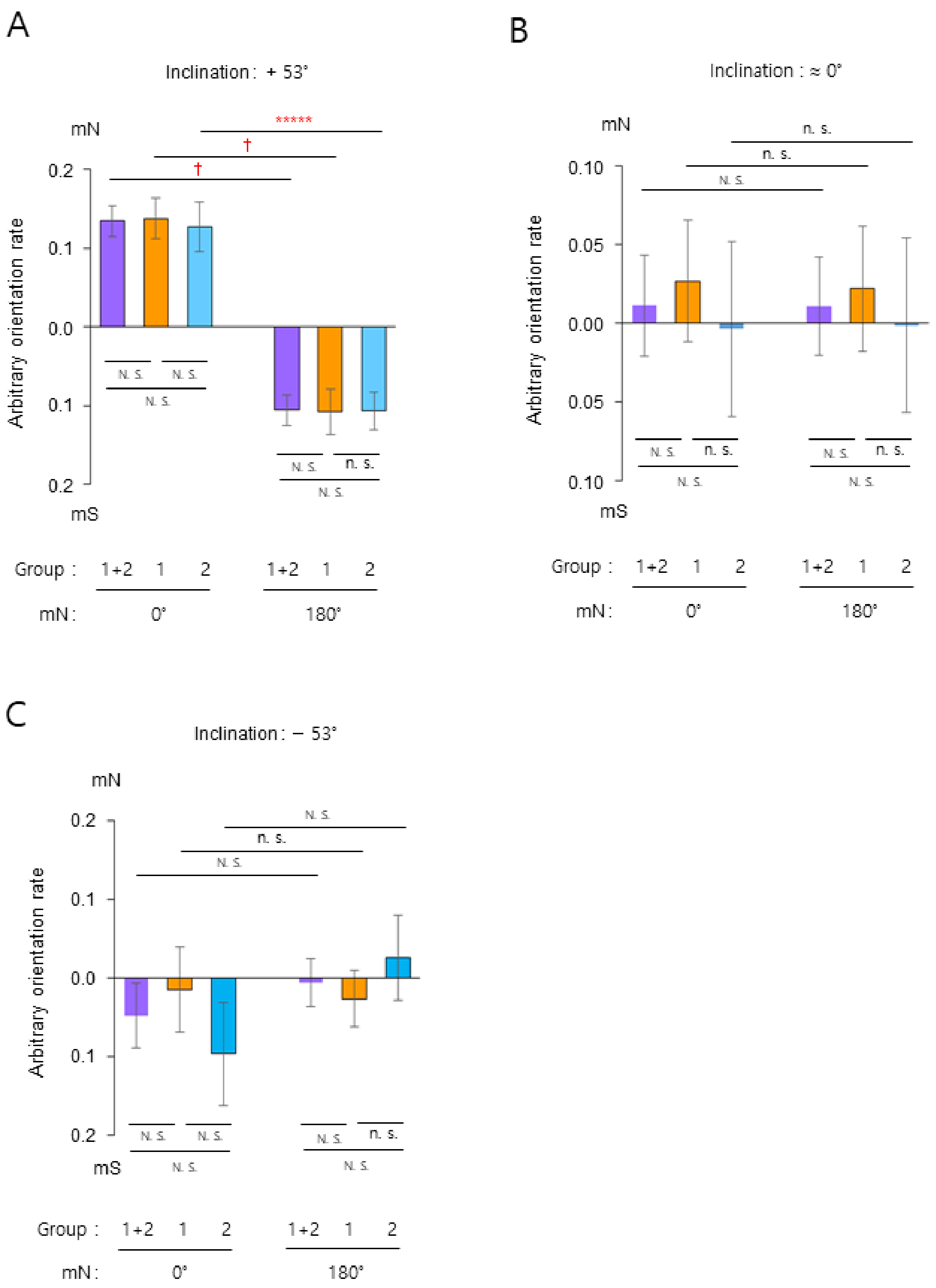
A
Inclination : + 53°
mN
mS
Arbitrary orientation rate
Group : 1+2 1 2 1+2 1 2
mN : 0° 180°
N. S.
n. s.
B
Inclination : ≈ 0°
C
Inclination : − 53°

Fig. S7. Magnetic orientation in groups under different inclinations.

Selected starved subjects were tested under different inclinations with the full wavelength of light and the orientation rate was presented by group. Note that the Y axis represents arbitrary orientation rate toward true magnetic north (mN) or true magnetic south (mS). **(A)** Inclination + 53° as the ambient GMF was maintained. **(B)** Inclination ≈ 0° due to a cancellation of the vertical component of the ambient GMF. **(C)** Inclination − 53° due to an inversion of the vertical component of the ambient GMF. *****, $P < 0.0005$; n.s. in the upper of the graph, not significant by paired sample *t*-test; n.s. in the lower of the graph, not significant by two-sample *t*-test; †, significant; N. S., not significant by a percentile bootstrap analysis (see Appendixes S1 and S2); error bars, standard error of the mean (SEM). Group 1, $n = 13$; Group 2, $n = 9$; Groups 1 + 2, $n = 22$.

Table S1. Experimental light conditions.

Filter goggles (#)	1	2	3	4	5	6	7	8	9	10
Color	UV-A-Red	Blue-Red	Green-Red	Yellow-Red	Orange-Red	Blue	Green-Yellow	Blue-Yellow	Green-Orange	UV-A
Light wavelength (nm) [a]	350-800 (350-800) [b]	400-800 (400-800)	491-800 (500-800)	569-800 (570-800)	595-800 (600-800)	400-505 (400-500)	500-573 (500-570)	400-573 (400-570)	500-608 (500-600)	350-405 (350-400)
Intensity of illumination (lux)	300.0	116.0	77.3	55.2	48.3	37.4	21.9	59.0	30.5	22.7
Light intensity (10^{13} x photons/cm^2/s)	18.2 (2.14) [c]	8.4 (0.99)	8.2 (0.96)	8.2 (0.96)	8.5 (1.00)	8.5 (1.00)	8.4 (0.99)	10.1 (1.19)	8.5 (1.00)	8.4 (0.99)

Subjects were provided with either full wavelength light or light filtered depending on experiments. Note that light intensity was measured on the surface of glabella (n = 2); the inner surfaces of the filter goggles were lightly sprayed with distilled water to mimic the moisturized conditions during experiments. For # 1, no goggles. [a] Light transmittance of > 50%, [b] values in parentheses in the same row present corresponding names of light wavelength mentioned in the main text, [c] values in the parentheses in the same row present relative light intensity compared to the intensity of blue light.

Table S2. Blood glucose levels in different experimental conditions.

Light wavelength (nm)	mN direction (°)	Starvation	Blood glucose concentration (mM)					
			Group 1			Group 2		
			#1 [a]	#2 [a]	#3	#1 [a]	#2 [a]	#3
350-800	0, 180	Y [b]	5.1±0.1	5.1±0.1	5.7±0.1	5.3±0.1	5.3±0.1	6.0±0.1
		N	6.5±0.2	6.7±0.1	6.6±0.2	6.2±0.2	6.5±0.2	6.4±0.2
350-800	90, 270	Y [b]	5.2±0.1	5.2±0.1	5.8±0.1	5.1±0.1	5.1±0.1	5.7±0.1
		N	6.4±0.2	6.3±0.2	6.8±0.3	6.3±0.3	6.4±0.3	6.4±0.2
400-800	0, 180	Y [b]	5.3±0.1	5.4±0.1	5.7±0.1	5.1±0.1	5.3±0.1	5.8±0.2
		N	6.3±0.3	6.6±0.2	6.7±0.2	6.7±0.3	6.9±0.3	7.0±0.3
500-800	0, 180	Y [b]	5.2±0.1	5.3±0.1	5.9±0.1	5.1±0.1	5.2±0.1	5.5±0.4
		N	6.6±0.2	6.7±0.2	6.8±0.2	6.5±0.3	6.8±0.3	6.6±0.3
570-800	0, 180	Y [b]	5.1±0.1	5.2±0.1	5.7±0.1	5.2±0.1	5.3±0.1	5.8±0.1
		N	6.3±0.3	6.5±0.3	6.7±0.2	6.4±0.3	6.5±0.3	6.8±0.2
600-800	0, 180	Y [b]	5.2±0.1	5.3±0.1	5.8±0.1	5.1±0.1	5.2±0.1	5.9±0.2
		N	6.5±0.2	6.7±0.2	6.8±0.3	6.3±0.3	6.6±0.2	6.9±0.3
350-400	0, 180	Y [b]	5.1±0.1	5.2±0.1	5.7±0.1	5.2±0.1	5.3±0.1	6.0±0.2
		N	6.4±0.2	6.6±0.2	6.8±0.3	N/A	N/A	N/A
400-500	0, 180	Y [c]	5.2±0.1	5.3±0.1	5.9±0.1	N/A	N/A	N/A
500-570	0, 180	Y [c]	5.1±0.1	5.2±0.1	5.8±0.1	N/A	N/A	N/A
400-570	0, 180	Y [c]	5.2±0.1	5.3±0.1	5.7±0.1	N/A	N/A	N/A
500-600	0, 180	Y [c]	5.2±0.1	5.4±0.1	5.9±0.1	N/A	N/A	N/A

Blood glucose levels during the 2-AFC experiments were determined at shortly before the first session (#1) and immediately after each session (#2 and #3). [a]$P < 0.001$ between

blood glucose concentration (BGC) of #1 and #3 or #2 and #3 in Group 1 and Group 2, [b]$P <$ 0.001 between corresponding BGC of #1s, #2s, or #3s under the Y and N experimental condition in Group 1 and Group 2, [c]$P <$ 0.001 between corresponding BGC of #1s, #2s, or #3s from the Ys and N in the 0, 180 of 350–800 nm condition by paired sample *t*-test. Statistical values are presented as mean ± standard error of the mean (SEM). mN direction is indicated as degree in clockwise direction from the ambient geomagnetic field direction. mN, magnetic north; Y, yes; N, no; N/A, not applicable. Group 1, $n = 20$ and Group 2, $n = 14$.

Table S3. Intensity of oscillating magnetic field on the glabella by the magnetic field angle relative to rotatory subject facing direction under the different mag netic field orientations.

	Angle (º)[a]		− 30	0	30	60	90	120	150	180	210
	(MHz)	(º)[b]									
MF intensity (nT)	1.260	0	48	41	48	49	60	76	97	100	97
	1.260	37	99	100	100	100	100	100	100	100	99
	1.260	74	97	100	97	76	60	49	48	41	48
	1.890	37	99	100	100	100	100	100	100	100	99
	0.060 –0.400	37	99	100	100	100	100	100	100	100	99

[a] clockwise angle of subject facing direction relative to the ambient magnetic north. [b] orientation of the oscillating magnetic field from antenna with respect to the ambient geomagnetic field. MF, magnetic field. Each intensity value was the mean from measurements on the three subjects with the highest, median, and the lowest sitting height on the chair.
